# Synthesis and Cytotoxic Activity of 1,3‐Selenazole‐2‐Amine Derivatives With Computational ADMET Profiling

**DOI:** 10.1002/open.70314

**Published:** 2026-10-01

**Authors:** Gulay Dilek, Senem Akkoc

**Affiliations:** ^1^ Faculty of Pharmacy Department of Basic Pharmaceutical Sciences Zonguldak Bulent Ecevit University Zonguldak Türkiye; ^2^ Faculty of Pharmacy Department of Basic Pharmaceutical Sciences Suleyman Demirel University Isparta Türkiye; ^3^ Faculty of Engineering and Natural Sciences Bahçeşehir University Istanbul Türkiye

**Keywords:** 1,3‐selenazole, antitumor agents, cytotoxicity, Hantzsch‐type cyclization, selenium

## Abstract

A set of newly developed 1,3‐selenazole‐2‐amine derivatives (**1–4**) was prepared via a modified Hantzsch‐type cyclization of appropriately substituted α‐bromoacetophenones with selenourea under reflux conditions. The synthesized compounds were tested for cytotoxic activity in HT‐29 (colon cancer), MCF‐7 (breast cancer), and L929 (mouse fibroblast) cell lines using the MTT assay. The compounds induced weak to moderate cytotoxicity in MCF‐7 cells. Among them, compound **4** showed cytotoxic activity with IC_50_ value of 171.10 µM, while no appreciable activity was observed against HT‐29 cells (IC_50_ > 300 µM). Selectivity analysis demonstrated that compound **3** exhibited low toxicity toward healthy cells. In silico ADMET analysis indicated promising pharmacokinetic and physicochemical properties, including high gastrointestinal absorption, moderate lipophilicity, and compliance with drug‐likeness rules, while predicting limited blood–brain barrier permeability. The BOILED‐Egg model further supported high intestinal absorption and peripheral activity.

## Graphical abstract

Four novel 1,3‐selenazole‐2‐amine derivatives were synthesized and evaluated in HT‐29, MCF‐7, and L929 cell lines. Compound **4** showed the highest MCF‐7 cytotoxicity. Compound **3** displayed low L929 toxicity. In silico ADMET predicted favorable pharmacokinetic properties.

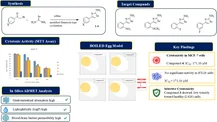

## 1 Introduction

Cancer continues to be a major burden on global health, contributing significantly to both morbidity and mortality rates [1]. Advancements in radiotherapy, chemotherapy, and targeted approaches have enhanced cancer treatment; however, therapeutic success is often hindered by the development of drug resistance and adverse systemic effects [2]. Therefore, the search for novel and more effective chemotherapeutic agents has become a major focus in current medicinal chemistry. In this context, heterocyclic scaffolds constitute a fundamental structural basis for the development of biologically active compounds [3]. Their structural diversity and tunable physicochemical properties make them ideal candidates for anticancer drug discovery [4].

Interest in organoselenium‐containing heterocycles has grown in recent years, mainly due to their wide range of biological activities. These compounds are known to display multiple pharmacological effects, including antibacterial [5], antifungal [6], antiparasitic [7, 8], antiviral [9], anti‐ischemic [10], antioxidant [11], anticancer [12, 13, 14], antidiabetic [15], antithyroid [16], and anti‐inflammatory [17] activities.

The remarkable biological potential of organoselenium heterocycles can be attributed to the unique chemical and redox properties of selenium itself. The growing interest in selenium chemistry originated from the discovery of selenocysteine, recognized as an additional proteinogenic amino acid beyond the standard 20, at the active site of glutathione peroxidase, an enzyme involved in antioxidant defense [18]. Selenium plays an essential role in regulating cellular redox balance through selenoproteins like glutathione peroxidase, thioredoxin reductase, and iodothyronine deiodinase, which collectively regulate oxidative stress and cellular defense mechanisms [19]. Selenium exhibits antimetastatic effects, particularly against breast, liver, prostate, melanoma, and lung cancers, and at low concentrations, it exerts protective effects against cancer development [20, 21, 22].

Replacing sulfur or oxygen atoms with selenium in bioactive molecules often enhances their redox and pharmacological properties, resulting in improved selectivity and efficacy against cancer cells and making organoselenium derivatives promising candidates for drug discovery [9, 13, 23]. Previous studies have shown that selenium‐containing analogs of classical heterocycles display markedly stronger antitumor activity compared with their sulfur or oxygen counterparts [23, 24, 25]. For example, selenazofurin, the selenium analog of the antitumor agent tiazofurin, has been reported to be 3–10‐fold more cytotoxic than tiazofurin against P388, HL‐60, and L1210 cells and to exhibit in vivo activity in P388 leukemia models, showing potent inhibition of inosine monophosphate dehydrogenase (IMPDH) types I and II [26, 27, 28] (Figure 1). Therefore, the introduction of selenium into heterocyclic frameworks has become an effective approach to improve biological activity and develop novel anticancer agents.

**FIGURE 1:**
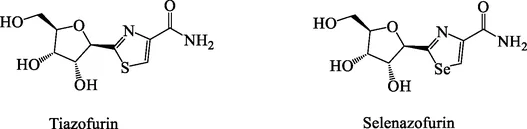
Structural comparison of thiazofurin and its selenium analog, selenazofurin, illustrating the substitution of sulfur with selenium in the heterocyclic ring.

Among organoselenium heterocycles, selenazoles have emerged as particularly promising scaffolds due to their potent anticancer activity [26, 29, 30, 31, 32]. However, the structure–activity relationship of selenazole derivatives in cancer models has not been fully explored. Within this framework, a novel set of selenazole derivatives was prepared, and their anticancer potential was examined in selected cancer cell lines.

## 2 Experimental Methods

### 2.1 Materials and Reagents

^1^H and ^13^C NMR spectra (600 and 150 MHz, respectively) were obtained on an Agilent Technologies 600 MHz Premium Compact NMR instrument. Tetramethylsilane (TMS) served as the internal reference. Spectra were recorded in DMSO‐d_6_. FTIR spectra were acquired with a Thermo Fisher Scientific Nicolet 6700 instrument fitted with an ATR module. Mass spectra were recorded using Shimadzu Nexera‐iLC‐2040C HPLC instrument. The melting points of the compounds were determined using a Electrothermal IA9200 melting point apparatus and no correction was applied. All reagents were purchased and used without further purification. 2‐Bromo‐1‐(2‐methoxyphenyl)ethan‐1‐one, 2‐bromo‐1‐(2‐nitrophenyl)ethan‐1‐one, 2‐bromo‐1‐(3‐fluorophenyl)ethan‐1‐one, and 2‐bromo‐1‐(2‐fluorophenyl)ethan‐1‐one were sourced from BLDpharm. Selenourea, sodium carbonate (Na_2_CO_3_), and methanol were purchased from Sigma‐Aldrich.

### 2.2 Synthesis of 3‐Selenazol‐2‐Amines

3‐Selenazol‐2‐amines (**1**–**4**) were synthesized via a modified Hantzsch‐type cyclization of appropriately substituted α‐bromoacetophenones with selenourea [33]. A suitably substituted α‐bromoacetophenone (0.0050 mol) and selenourea (0.92 g, 0.0075 mol) were dissolved in 5 mL of methanol, and the mixture was then heated under reflux until no starting material remained, as confirmed by thin‐layer chromatography (TLC). Following completion of the reaction, the mixture was cooled to ambient temperature and then poured into a 5% aqueous sodium carbonate solution (20 mL). A solid formed, which was filtered, washed with water, and dried under vacuum.

#### 2.2.1 4‐(2‐Nitrophenyl)‐1,3‐Selenazol‐2‐Amine (1)

2‐Bromo‐1‐(2‐nitrophenyl)ethan‐1‐one (1.22 g, 0.0050 mol) was used as the starting material to afford 4‐(2‐nitrophenyl)‐1,3‐selenazole‐2‐amine. Red solid; yield: 83%; m.p.: 117–118 °C. IR (ATR, cm^−1^): 3413, 3378 (N–H), 3091 (Ar–H), 1631 (C=N), 1599 (NO_2_ asym), 1509 (Ar C=C), 1360 (NO_2_ sym), 1326, 1292, 1187, 1127 (C–N), 679, 635 (C–Se). ^1^H NMR (600 MHz, DMSO‐d_6_, 298 K) δ ppm: 7.75–7.45 (m, 4H, Ar–H), 7.30 (s, 1H, selenazole C5–H), 7.25 (br s, 2H, NH_2_). ^13^C APT (150 MHz, DMSO‐d_6_, 298 K) δ ppm: 170.1 (selenazole C2), 149.3 (CAr–NO_2_), 147.3 (selenazole C4), 132.5, 130.8 (CAr–H), 130.2 (ipso‐CAr), 128.8 (selenazole C5), 123.9, 109.9 (CAr–H). HRMS (ESI^+^) m/z: calcd for C_9_H_7_N_3_O_2_SeNa^+^ [M + Na]^+^ 291.9596; found 291.9600.

#### 2.2.2 4‐(2‐Methoxyphenyl)‐1,3‐Selenazol‐2‐Amine (2)

2‐Bromo‐1‐(2‐methoxyphenyl)ethan‐1‐one (1.14 g, 0.0050 mol) was used as the starting material to afford 4‐(2‐methoxyphenyl)‐1,3‐selenazole‐2‐amine. Red solid; yield: 65%; m.p.: 96–97 °C. IR (ATR, cm^−1^): 3435, 3279 (N–H), 3112 (Ar–H), 2956 (C–H), 1644 (C=N), 1597, 1532, 1468 (Ar C=C), 1319, 1284 (C–N), 1239 (C–O), 635 (C–Se). ^1^H NMR (600 MHz, DMSO‐d_6_, 298 K) δ ppm: 8.00 (d, *J* = 8.2 Hz, 1H, Ar–H), 7.60 (s, 1H, selenazole C5–H), 7.25–7.18 (m, 1H, Ar–H), 7.16 (br s, 2H, NH_2_), 7.00 (d, *J* = 8.2 Hz, 1H, Ar–H), 6.91 (t, *J* = 7.6 Hz, 1H, Ar–H), 3.82 (s, 3H, OCH_3_). ^13^C APT (150 MHz, DMSO‐d_6_, 298 K) δ ppm: 167.7 (selenazole C2), 156.9 (CAr–OCH_3_), 147.0 (selenazole C4), 130.4, 128.3 (CAr–H), 124.4 (ipso‐CAr), 120.7 (selenazole C5), 111.8, 110.8 (CAr–H), 55.7 (OCH_3_). HRMS (ESI^+^) m/z: calcd for C_10_H_11_N_2_OSe^+^ [M + H] ^+^ 255.0037; found 255.0057.

#### 2.2.3 4‐(2‐Fluorophenyl)‐1,3‐Selenazol‐2‐Amine (3)

2‐Bromo‐1‐(2‐fluorophenyl)ethan‐1‐one (1.08 g, 0.0050 mol) was used as the starting material to afford 4‐(2‐fluorophenyl)‐1,3‐selenazole‐2‐amine. Yellow solid; yield: 86%; m.p.: 138–139 °C. IR (ATR, cm^−1^): 3420, 3219 (N–H), 3137, 3079 (Ar–H), 1590 (C=N), 1530, 1506, 1468, 1447 (Ar C=C), 1323, 1288, 1256 (C–N), 1213, 1171, 1104 (C–F), 669, 637 (C–Se). ^1^H NMR (600 MHz, DMSO‐d_6_, 298 K) δ ppm: 8.00 (d, *J* = 7.8 Hz, 1H, Ar–H), 7.42 (br s, 2H, NH_2_), 7.30 (s, 1H, selenazole C5–H), 7.29–7.23 (m, 2H, Ar–H), 7.24–7.10 (m, 1H, Ar–H). ^13^C APT (150 MHz, DMSO‐d_6_, 298 K) δ ppm: 169.2 (selenazole C2), 160.7 (CAr–F), 144.8 (selenazole C4), 130.6, 128.9 (CAr–H), 124.8 (selenazole C5), 123.6 (ipso‐CAr), 116.2, 111.2 (CAr–H). HRMS (ESI^+^) m/z: calcd for C_9_H_7_FN_2_SeNa^+^ [M + Na]^+^ 264.9650; found 264.9670.

#### 2.2.4 4‐(3‐Fluorophenyl)‐1,3‐Selenazol‐2‐Amine (4)

2‐Bromo‐1‐(3‐fluorophenyl)ethan‐1‐one (1.08 g, 0.0050 mol) was used as the starting material to afford 4‐(3‐fluorophenyl)‐1,3‐selenazole‐2‐amine. Yellow solid; yield: 87%; m.p.: 112–113 °C. IR (ATR, cm^−1^): 3422, 3260 (N–H), 3111 (Ar–H), 1584 (C=N), 1528, 1484, 1439 (Ar C=C), 1308, 1288, 1265 (C–N), 1144 (C–F), 632 (C–Se). ^1^H NMR (600 MHz, DMSO‐d_6_, 298 K) δ ppm: 7.61 (d, *J* = 7.7 Hz, 1H, Ar–H), 7.56 (s, 1H, selenazole C5–H), 7.54–7.50 (m, 1H, Ar–H), 7.37–7.33 (m, 1H, Ar–H), 7.32 (br s, 2H, NH_2_), 7.05–7.00 (m, 1H, Ar–H). ^13^C APT (150 MHz, DMSO‐d_6_, 298 K) δ ppm: 169.8 (selenazole C2), 163.7 (CAr–F), 149.8 (selenazole C4), 138.5 (ipso‐CAr), 130.7, 122.2 (CAr–H), 114.0 (selenazole C5), 112.8, 107.9 (CAr–H). HRMS (ESI^+^) m/z: calcd for C_9_H_8_FN_2_Se^+^ [M + H] ^+^ 242.9837; found 242.9863.

### 2.3 In Vitro Cytotoxicity Studies

The human colorectal cancer (HT‐29), human breast cancer (MCF‐7), and mouse fibroblast (L929) cell lines were obtained from the American Type Culture Collection (ATCC, USA). Cells were maintained in their respective culture media supplemented with fetal bovine serum (FBS) and GlutaMAX (RPMI for MCF‐7, McCoy’s 5A for HT‐29, and EMEM for L929) and grown in 25 cm^2^ culture flasks. The cells were initially cultured in 25 cm^2^ culture flasks; after reaching sufficient density, they were detached from the surface using trypsin‐EDTA, transferred to 75 cm^2^ flasks, and continued to be cultured to increase the cell count. Cells were plated in sterile 96‐well plates at a density of 5 × 10^3^ cells per well [34]. After 24 h of incubation, the cells were treated with the compounds at concentrations of 300, 150, 75, and 37.5 µM. The exposure period was 48 h for HT29 cells and 72 h for MCF‐7 and L929 cells. After the incubation period was over, the culture medium was carefully removed from the wells. Then, 50 µL of MTT stock solution (5 mg/mL) was added to wells, and incubated for 2 h. After the 2‐h incubation period had expired, the culture medium was carefully removed from each well, and 200 μL of DMSO was added to the medium to dissolve the formed formazan. The plates were left to mix in the dark for half an hour. The absorbance values were measured by the BioTech Elisa plate reader at 630 nm. IC_50_ values were calculated using GraphPad Prism software 5. Each experiment was carried out independently in three replicates, and the data are presented as mean values derived from two independent experiments.

### 2.4 ADME and Toxicity (ADMET) Studies

The SMILES representation of the compound **1**, NC1=NC(C2=CC=CC=C2[N+]([O‐])=O)=C[Se]1, compound **2**, NC1=NC(C2=CC=CC=C2OC)=C[Se]1, compound **3**, NC1=NC(C2=CC=CC=C2F)=C[Se]1, compound **4**, NC1=NC(C2=CC=CC(F)=C2)=C[Se]1, were generated using ChemBioDraw Ultra 15.0 software. The codes based on these structure served as input for the in silico assessment of pharmacokinetic and toxicity profiles. ADME‐related parameters were predicted using the SwissADME online tool [35], toxicity assessments were performed using the ProTox‐II platform [36].

## 3 Results and Discussion

### 3.1 Chemistry

1,3‐Selenazol‐2‐amine derivatives (**1–4**) were successfully synthesized via a Hantzsch‐type cyclization between appropriately substituted 2‐bromoacetophenones and selenourea in methanol (Scheme 1). The reactions proceeded smoothly under reflux conditions, affording the target compounds in good yields (65%–87%).

**SCHEME 1:**
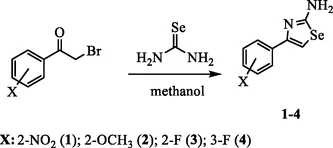
Synthesis of selenazoles (**1–4**).

The formation of the 1,3‐selenazole core is consistent with a typical nucleophilic substitution–cyclization mechanism. Initially, the selenium atom of selenourea attacks the electrophilic α‐carbon of the corresponding bromoacetophenone, leading to the formation of a selenoimidate intermediate. This intermediate subsequently undergoes intramolecular cyclization, followed by elimination of hydrogen bromide, yielding the desired 1,3‐selenazole derivatives (Scheme 2). A similar mechanistic pathway has been reported for related heterocyclic systems (e.g., [37]).

**SCHEME 2:**
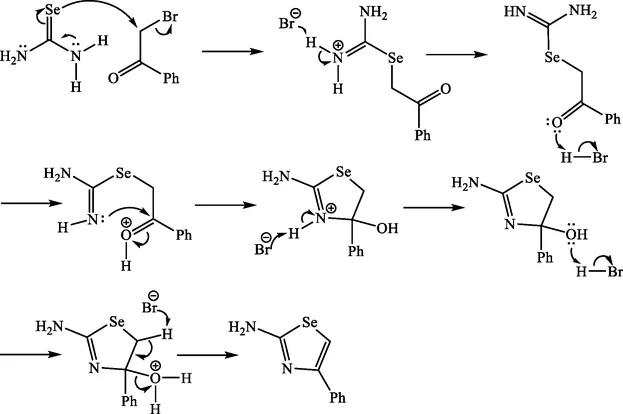
Proposed mechanism for the synthesis of 1,3‐selenazol‐2‐amines via nucleophilic substitution and intramolecular cyclization between α‐bromoacetophenones and selenourea.

Based on the isolated yields, substrates bearing electron‐withdrawing groups (e.g., nitro and fluoro) afforded higher yields (83%–87%), whereas the methoxy‐substituted derivative gave a lower yield (65%). This observation may be rationalized by the increased electrophilicity of the α‐carbon in bromoacetophenones bearing electron‐withdrawing substituents, which facilitates nucleophilic attack by selenourea.

Structural characterization of the synthesized compounds was performed by means of IR, ^1^H NMR, ^13^C NMR, and HRMS. In the IR spectra, characteristic N–H stretching bands were observed in the range of 3200–3450 cm^−1^, while the C=N stretching vibrations appeared around 1580–1650 cm^−1^. In the ^1^H NMR spectra, a singlet at 7.25–7.60 ppm was assigned to the C5–H proton of the selenazole ring, while the NH_2_ protons appeared as singlets in the 7.16–7.42 ppm region. Aromatic protons appeared as multiplets in the range of 7.00–8.00 ppm, depending on the substitution pattern of the phenyl ring. For compound **2**, a singlet observed at 3.82 ppm was assigned to the methoxy (–OCH_3_) group. The ^13^C APT spectra supported the proposed structures. The C2 carbon resonated in the range of δ 167–170 ppm, while the C4 carbon was observed at δ 145–150 ppm. The C5 carbon appeared at δ 114–129 ppm, depending on the nature of the aryl substituent. The HRMS data were in good agreement with the calculated values, supporting the proposed molecular structures. To the best of our knowledge, compounds **1–4** have not been previously reported.

### 3.2 Cytotoxicity Studies

The cytotoxic activities of the synthesized 1,3‐selenazole‐2‐amine derivatives (**1**–**4**) were evaluated against MCF7, HT29, and L929 cell lines (Table 1). Compounds **2**–**4** exhibited moderate to weak cytotoxic activity against the MCF7 cell line, with IC_50_ values of 190.10, 230.50, and 171.10 µM, respectively, whereas compound **1** showed weak activity (IC_50_ = 315.80 µM). In contrast, all compounds showed no appreciable cytotoxicity against HT‐29 cells (IC_50_ > 300 µM), indicating a clear cell line‐dependent response. Notably, compound **2** displayed the highest toxicity toward L929 cells (IC_50_ = 77.11 µM), while the remaining compounds showed weaker or negligible effects.

**TABLE 1:** In vitro cytotoxicity (IC_50_, µM) of compounds 1–4 against human cancer and mouse fibroblast cell lines.

Compounds	**IC**_**50**_, **µM**		
	MCF7	HT29	L929
**1**	>300	>300	274.50
**2**	190.10	>300	77.11
**3**	230.50	>300	>300
**4**	171.10	>300	168.60
**Cisplatin**	13.96	N.T.*	19.44

*Note:* N.T.*, Not tested.

Among the tested compounds, compound **4** (3‐fluorophenyl) displayed the highest activity against MCF7 cells (IC_50_ = 171.10 µM), followed by compound **2** (2‐methoxyphenyl) and compound **3** (2‐fluorophenyl). These results suggest that both the nature and position of substituents on the phenyl ring influence cytotoxic activity. In particular, the meta‐fluoro substituted derivative (compound **4**) exhibited higher activity than its ortho‐fluoro counterpart (compound **3**), indicating that positional effects play a critical role. In contrast, the ortho‐nitro substituted compound (**1**) displayed very weak activity. Evaluation against the L929 healthy cell line revealed differences in cytotoxic selectivity. Compound **3** exhibited the lowest toxicity (IC_50_ = >300 µM). In contrast, compound **2** showed higher toxicity toward healthy cells (IC_50_ = 77.11 µM).

Compared with previously reported chromone‐based 1,3‐selenazole derivatives, which exhibited substituent‐dependent variations in cytotoxic activity against MCF‐7 cells (IC_50_ = 7.2–218.1 µg/mL, corresponding approximately to 17.8–595.0 µM across the series) [38], the compounds synthesized in the present study displayed generally weaker cytotoxic activity but a more uniform and cell line‐dependent response profile. This difference may be associated with the absence of the chromone moiety and differences in substituent patterns, suggesting that structural features play an important role in modulating cytotoxic potency. In addition, previously reported 1,3‐selenazole derivatives have demonstrated potent cytotoxic activity in different cancer cell lines, such as HT‐1080 fibrosarcoma cells, with IC_50_ values in the low micromolar range (2.5–4.5 µM) [39]. Although direct comparison is limited due to differences in cell line models, these findings demonstrate the potential of the 1,3‐selenazole scaffold as a promising pharmacophore for anticancer drug development.

The changes in cell viability resulting from the different concentrations of the compounds are presented in Figure 2. While no clear concentration‐dependent effect was observed in HT‐29 cells, a distinct concentration‐dependent decrease in cell viability was noted in MCF‐7 cells, with more pronounced effects at higher concentrations (150–300 µM). When healthy cells were evaluated, it was observed that compound **2**, in particular, exhibited a concentration‐dependent toxic effect. In contrast, no significant changes were detected in compounds **1** and **3** at concentrations of 37.5, 75, and 150 µM against the L929 cell line; however, a notable decrease in cell viability was detected only at a concentration of 300 µM.

**FIGURE 2:**
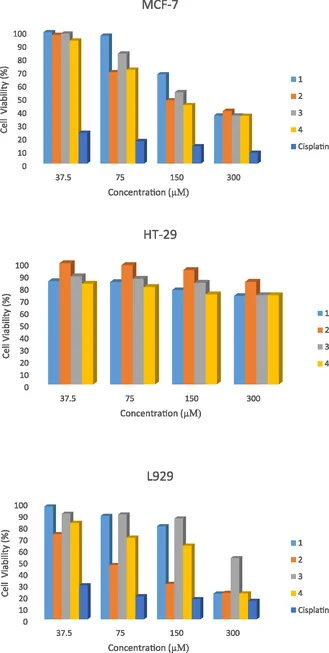
The cell viability ratio depending on tested compounds.

The selectivity index (SI) is an important parameter that reflects a compound’s efficacy against cancer cells relative to its toxicity toward healthy cells SI is calculated by dividing the IC_50_ value obtained in healthy cells by that in cancer cells (SI = IC_50_, healthy/IC_50_, cancer). A SI value greater than 1 indicates preferential cytotoxicity toward cancer cells. Based on these results, compound **3** exhibited the highest selectivity index (Table 2), reflecting its relatively low toxicity toward L929 cells compared with its moderate activity against MCF‐7 cells. In contrast, compound **2** showed a low selectivity index, indicating higher toxicity toward healthy cells relative to its anticancer activity.

**TABLE 2:** Selectivity index (SI) values for compounds 1–4 (L929 vs MCF‐7).

Compound	SI
**1**	0.87
**2**	0.41
**3**	2.13
**4**	0.99

MCF‐7 cells were imaged using a Leica inverted microscope after 72 h of incubation at concentrations of 37.5 and 75 µM (Figure 3). The images revealed a reduction in cell viability relative to the negative control, which was more pronounced at 75 µM.

**FIGURE 3:**
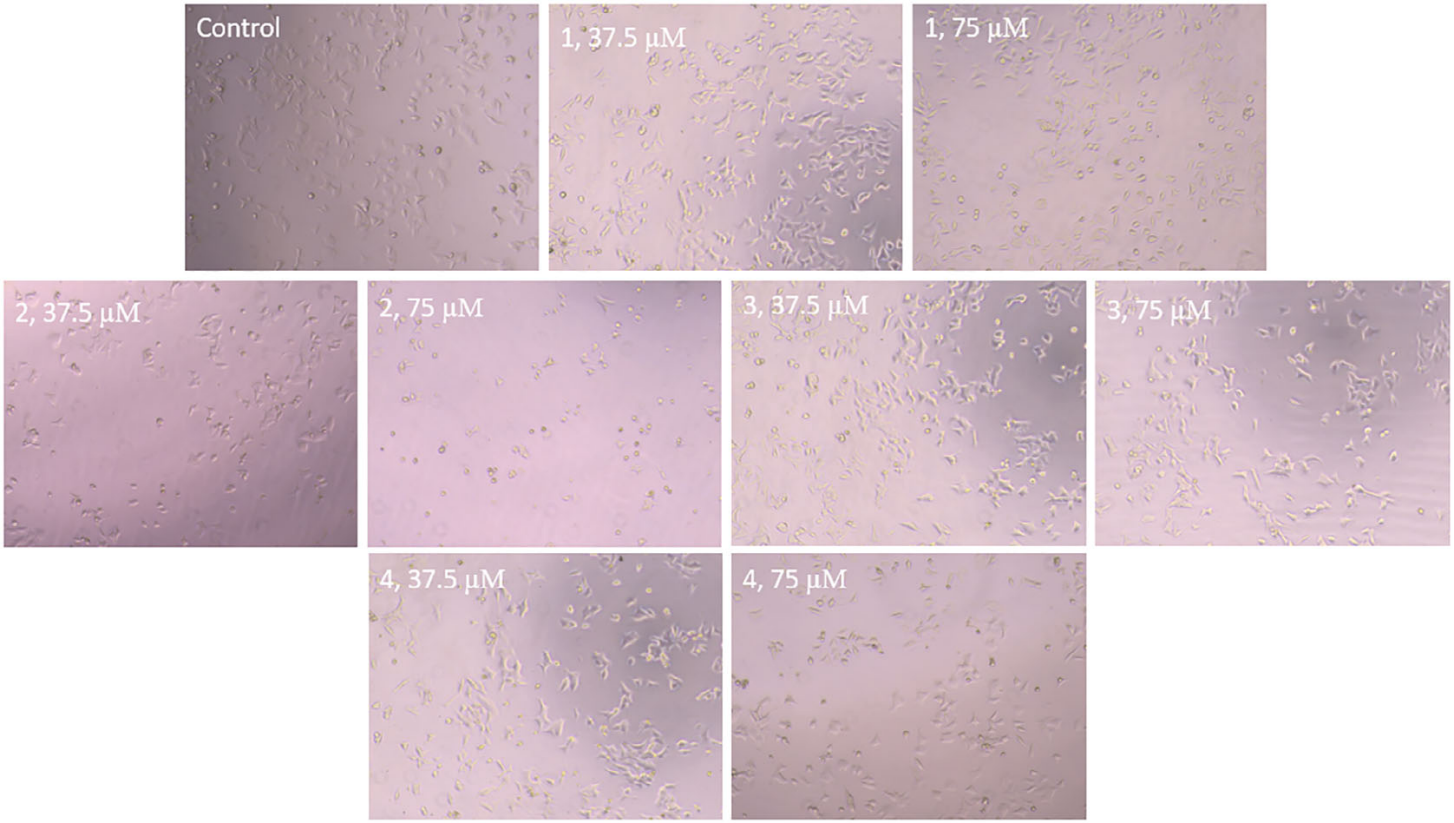
Inverted microscopy images of MCF‐7 cells after 72 h treatment with compound **4** at 37.5 and 75 µM compared with untreated control cells. Images were acquired using a Leica inverted microscope at 10× magnification. Scale bar: 75 µm.

### 3.3 ADME and Toxicity (ADMET) Properties of Compounds

Oral toxicity prediction results for compounds **1–4** are depicted in Figure 4. Compound **1** yielded an estimated LD_50_ value of 1230 mg/kg, suggesting relatively low acute toxicity. Its classification in toxicity class 4 further supports a low‐to‐moderate toxicity profile. Similarly, compound **2** showed an LD_50_ value of 1000 mg/kg, indicating a comparable toxicity profile. In contrast, compound **3** displayed a lower LD_50_ value of 720 mg/kg, suggesting a higher toxicity potential than compounds **1** and **2**, although it remains within toxicity class 4. Compound **4** exhibited the lowest LD_50_ value (150 mg/kg), indicating higher acute toxicity among the tested compounds, which is consistent with its classification in toxicity class 3.

**FIGURE 4:**
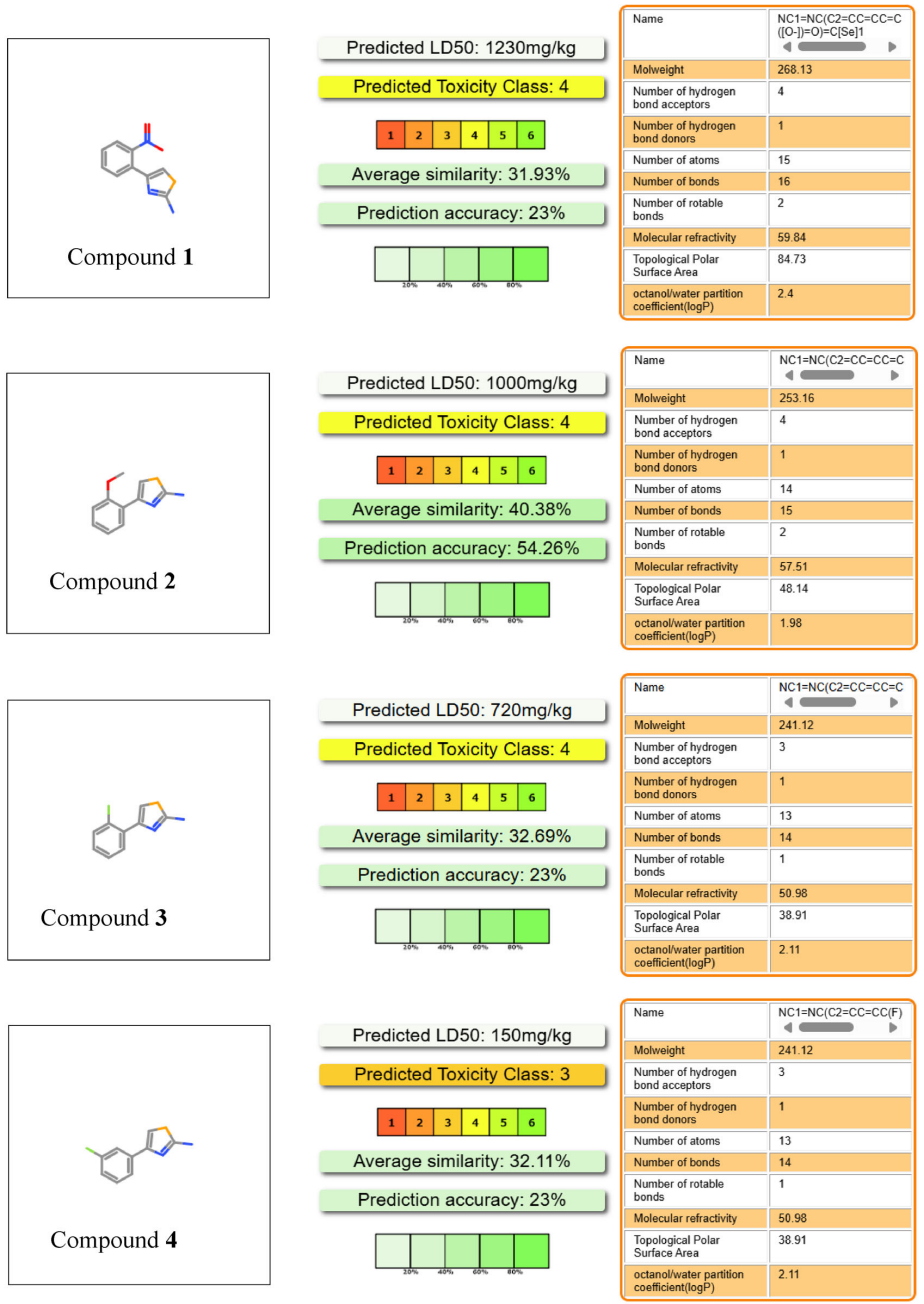
In silico assessment of oral toxicity profiles of compounds **1–4**.

According to the in silico analysis, the lower TPSA values observed for compounds **3** and **4** (≈39 Å^2^) are consistent with improved membrane permeability (Table 3). The consensus LogP values of the compounds ranged from −0.05 to 0.96, indicating moderate lipophilicity and a balanced profile favorable for oral absorption. Notably, compounds **3** and **4** exhibited relatively higher lipophilicity compared with the other derivatives, which may further contribute to their membrane permeability.

**TABLE 3:** In silico predicted pharmacokinetic, physicochemical, and drug‐likeness properties for compounds 1–4.

Physicochemical properties				
**Compounds**	**1**	**2**	**3**	**4**
Molecular formula	C_9_H_7_N_3_O_2_Se	C_10_H_10_N_2_OSe	C_9_H_7_FN_2_Se	C_9_H_7_FN_2_Se
Molecular weight (MW, g/mol)	268.13	253.16	241.12	241.12
Number of heavy atoms	15	14	13	13
Number of aromatic heavy atoms	11	11	11	11
Number of rotatable bonds	2	2	1	1
Number of H‐bond acceptors	3	2	2	2
Number of H‐bond donors	1	1	1	1
Molar refractivity	59.84	57.51	50.98	50.98
TPSA, Å^2^	84.73	48.14	38.91	38.91
**Lipophilicity**				
Log *P*_o/w_ (iLOGP)	0.00	0.00	0.00	0.00
Log *P*_o/w_ (XLOGP3)	1.05	1.19	1.32	1.32
Log *P*_o/w_ (WLOGP)	−0.40	−0.30	0.25	0.25
Log *P*_o/w_ (MLOGP)	0.24	1.02	1.75	1.75
Log *P*_o/w_ (SILICOS‐IT)	−1.13	1.06	1.50	1.50
Consensus log *P*_o/w_	−0.05	0.59	0.96	0.96
**Pharmacokinetics**				
GI absorption	High	High	High	High
BBB permeant	No	No	No	No
P‐gp substrate	No	No	No	No
CYP2C19 inhibitor	No	No	No	No
CYP3A4 inhibitor	No	Yes	No	No
CYP1A2 inhibitor	Yes	No	Yes	Yes
CYP2D6 inhibitor	No	No	No	No
CYP2C9 inhibitor	No	No	No	No
Log *K*_p_ (skin permeation, cm/s)	−7.19	−7.00	−6.83	−6.83
**Water solubility**				
Log S (ESOL) Class	−2.57 Soluble	−2.61 Soluble	−2.73 Soluble	−2.73 Soluble
Log S (Ali) Class	−2.42 Soluble	−1.80 Very soluble	−1.74 Very soluble	−1.74 Very soluble
Log S (SILICOS‐IT) Class	−3.00 Soluble	−3.75 Soluble	−3.90 Soluble	−3.90 Soluble
**Druglikeness**				
Lipinski	Yes; 0 violation	Yes; 0 violation	Yes; 0 violation	Yes; 0 violation
Ghose	No; 1 violation: WLOG*P* < −0.4	Yes	Yes	Yes
Veber	Yes	Yes	Yes	Yes
Egan	Yes	Yes	Yes	Yes
Muegge	Yes	Yes	Yes	Yes
Bioavailability score	0.55	0.55	0.55	0.55

When the pharmacokinetic properties of the synthesized compounds were evaluated, all compounds were predicted to exhibit high gastrointestinal absorption (HIA). None of the compounds were predicted to be blood–brain barrier (BBB) permeant, suggesting a low likelihood of central nervous system exposure. In addition, none of the compounds were identified as P‐gp substrates, indicating that P‐gp‐mediated efflux is unlikely. Evaluation of cytochrome P450 interactions revealed that compounds **1**, **3**, and **4** may inhibit CYP1A2, suggesting a potential for drug–drug interactions, while compound **2** was predicted to inhibit CYP3A4 (Table 3).

According to the predicted water solubility results, all compounds were classified as either soluble or highly soluble. Notably, based on the Ali model, compounds **2**, **3**, and **4** were categorized as highly soluble. Drug‐likeness evaluation showed that all compounds satisfied Lipinski, Veber, Egan, and Muegge criteria, supporting their potential as orally active drug candidates. In addition, a bioavailability score of 0.55 for all compounds indicates moderate oral bioavailability. The BOILED‐Egg model for compounds **1–4** is presented in Figure 5.

**FIGURE 5:**
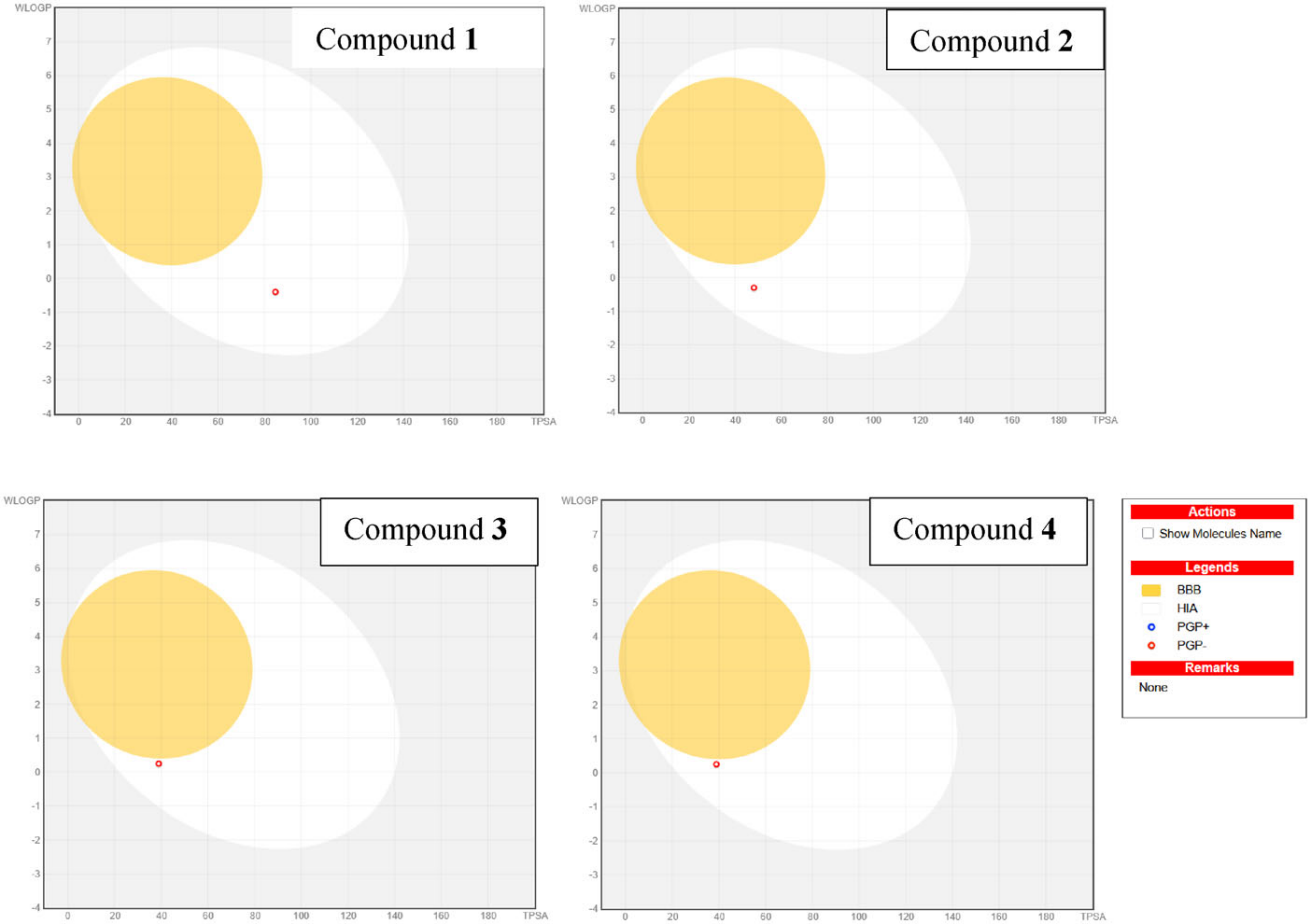
Boiled‐egg model of compounds **1–4**.

The BOILED‐Egg model offers a quick computational assessment of HIA and BBB penetration based on lipophilicity (WLOGP) and topological polar surface area (TPSA). In this model, the white region corresponds to high intestinal absorption, while the yellow region (yolk) indicates BBB permeability. All compounds are located within the white region. However, none of the compounds fall within the yolk region, indicating a low probability of BBB penetration (Figure 5). Notably, compounds **3** and **4** are positioned closer to the boundary of the yolk region compared with compounds **1** and **2**. This positioning suggests that their physicochemical properties, particularly lipophilicity and polarity, are relatively more balanced in terms of membrane permeability. In contrast, compounds **1** and **2** are located farther from the yolk region, indicating a lower tendency toward BBB permeation. Nevertheless, all compounds remain outside the BBB‐permeable region, confirming that their overall profiles are more consistent with peripheral rather than central activity.

## 4 Conclusion

In this study, a series of novel 1,3‐selenazole‐2‐amine derivatives (**1**‐**4**) were successfully synthesized via a modified Hantzsch‐type cyclization of appropriately substituted α‐bromoacetophenones with selenourea. The cytotoxic evaluation revealed that the compounds exhibited weak activity against MCF‐7 cells and negligible activity against HT‐29 cells, indicating a clear cell line‐dependent response. In silico ADMET analysis indicated that all compounds possess favorable physicochemical and pharmacokinetic properties, including high gastrointestinal absorption, moderate lipophilicity, and good drug‐likeness profiles. However, none of the compounds were predicted to penetrate the blood–brain barrier, suggesting a lower likelihood of central nervous system‐related effects. Furthermore, the BOILED‐Egg model supported these findings, indicating high intestinal absorption and limited BBB permeability for all compounds.

## Funding

This work was supported by the Süleyman Demirel Üniversitesi (TSG‐2024‐9516).

## Conflicts of Interest

The authors declare no conflicts of interest.

## Supporting information

Supplementary Material

## Data Availability

The data that support the findings of this study are available from the corresponding author upon reasonable request.
