# Supplementary material for: Synthesis and Cytotoxic Activity of 1,3‐Selenazole‐2‐Amine Derivatives With Computational ADMET Profiling
Source: ChemistryOpen. 2026 Oct 1;15(10):e70314. doi: 10.1002/open.70314 (PMC13631558; doi:10.1002/open.70314)
Supplement: Supplementary file 1 [file OPEN-15-e70314-s001.pdf]

SUPPORTING INFORMATION

Synthesis and Cytotoxic Activity of 1,3-Selenazole-2-Amine Derivatives with Computational ADMET Profiling

Gulay Dilek[1*], Senem Akkoc[2,3*],

[1]*Zonguldak Bulent Ecevit University, Faculty of Pharmacy, Department of Basic Pharmaceutical Sciences, Zonguldak, 67100, Türkiye*

[2]*Suleyman Demirel University, Faculty of Pharmacy, Department of Basic Pharmaceutical Sciences, Isparta, 32260, Türkiye.*

[3]*Bahçeşehir University, Faculty of Engineering and Natural Sciences, Istanbul, 34353, Türkiye.*

∗Corresponding author. Tel.: +90-372 2613154 (for GD); +90-246 2110334 (for SA).

E-mail addresses*:* gulay.dilek@beun.edu.tr (GD); senemakkoc@sdu.edu.tr; senemakkoc44@gmail.com (SA).

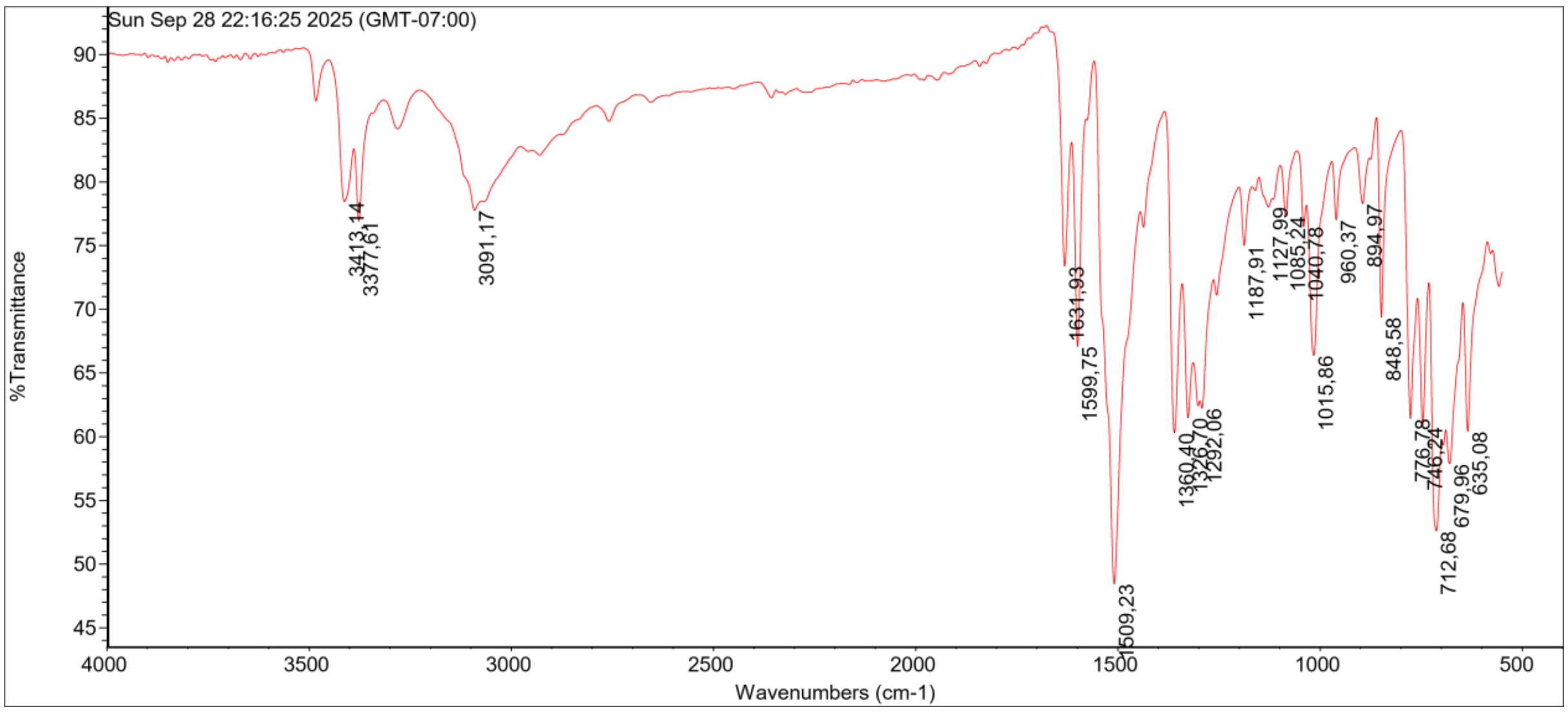

Figure S1. ATR-IR spectrum of 4-(2-nitrophenyl)-1,3-selenazol-2-amine (compound **1**)

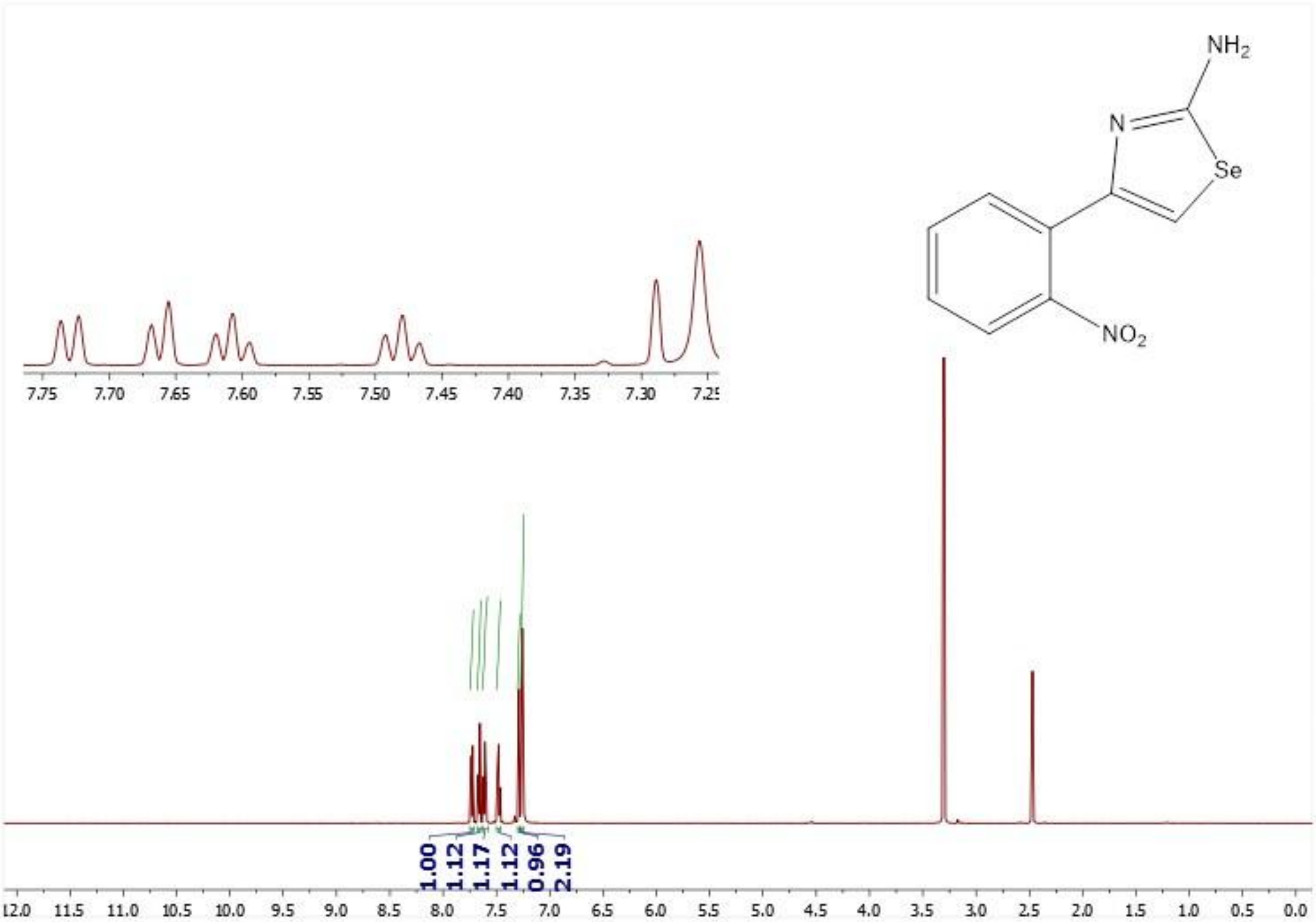

Figure S2. ^{1}H NMR spectrum of 4-(2-nitrophenyl)-1,3-selenazol-2-amine (compound **1**)

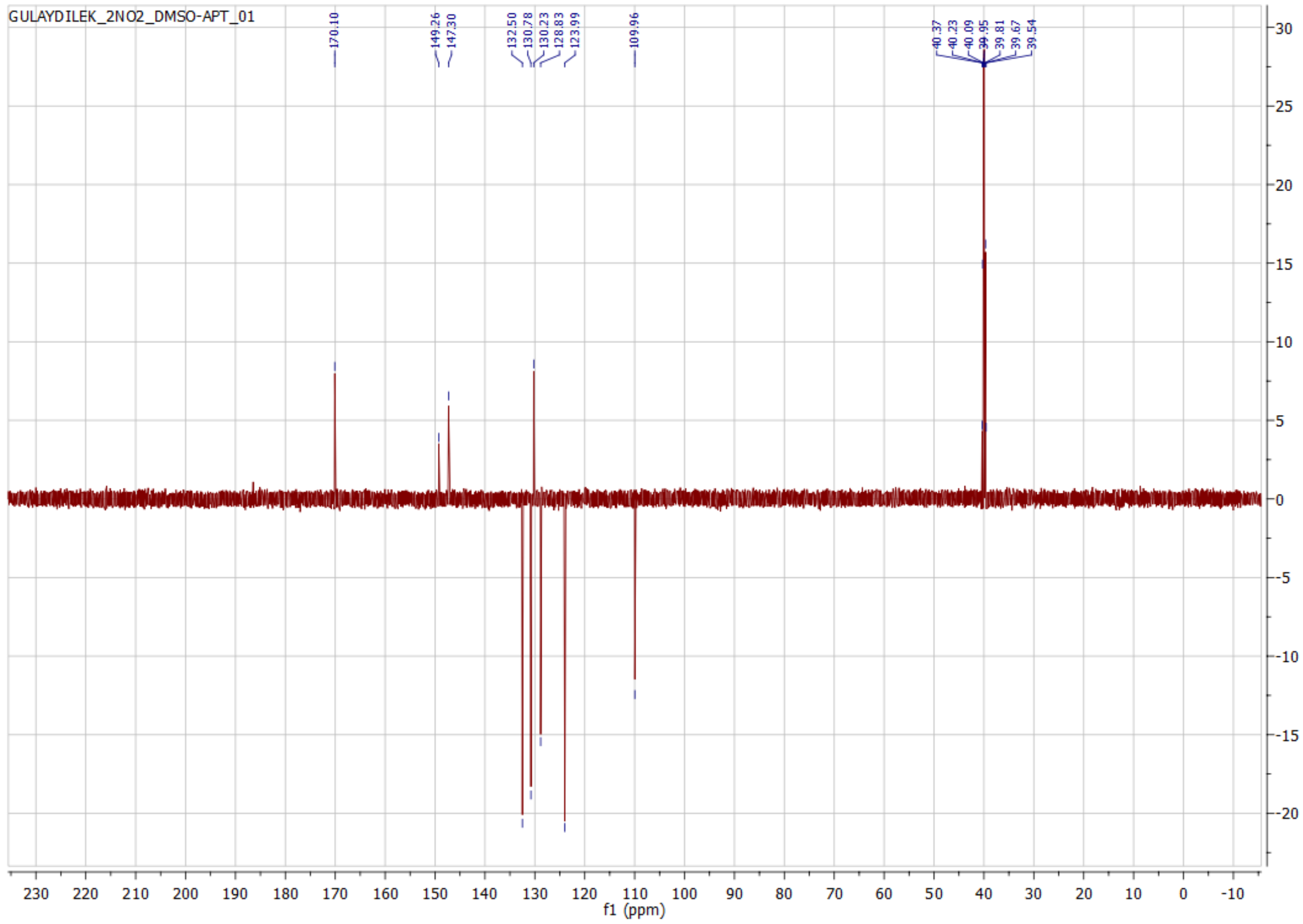

Figure S3. ^{13}C APT NMR spectrum of 4-(2-nitrophenyl)-1,3-selenazol-2-amine (compound **1**)

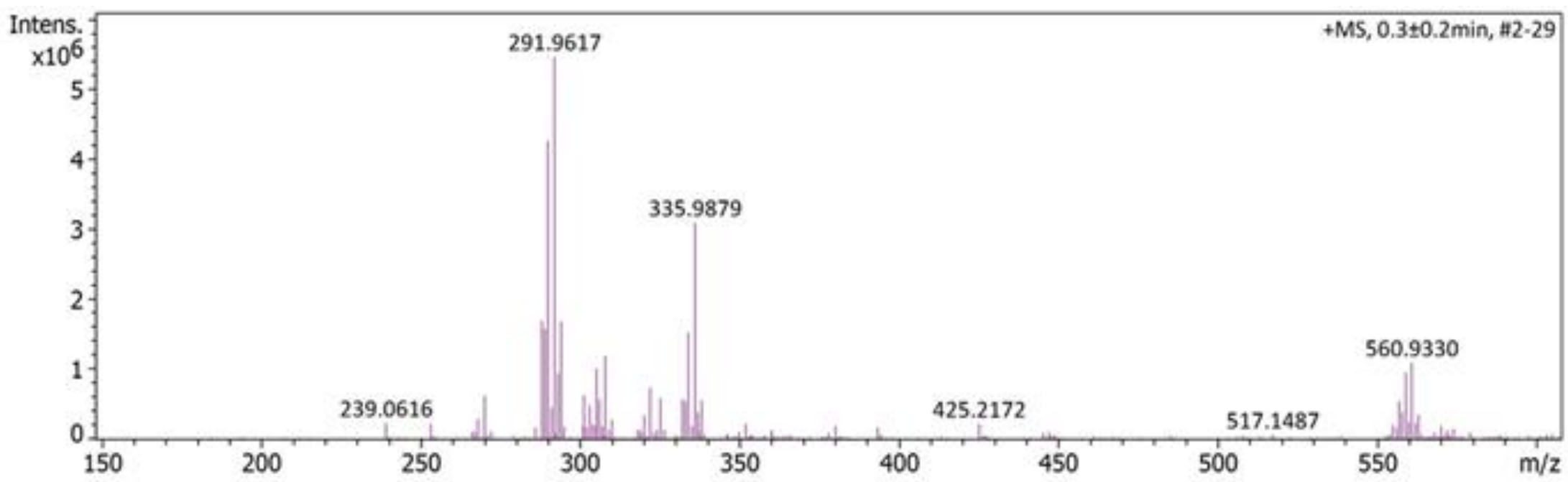

Figure S4. HRMS spectrum of 4-(2-nitrophenyl)-1,3-selenazol-2-amine (compound **1**)

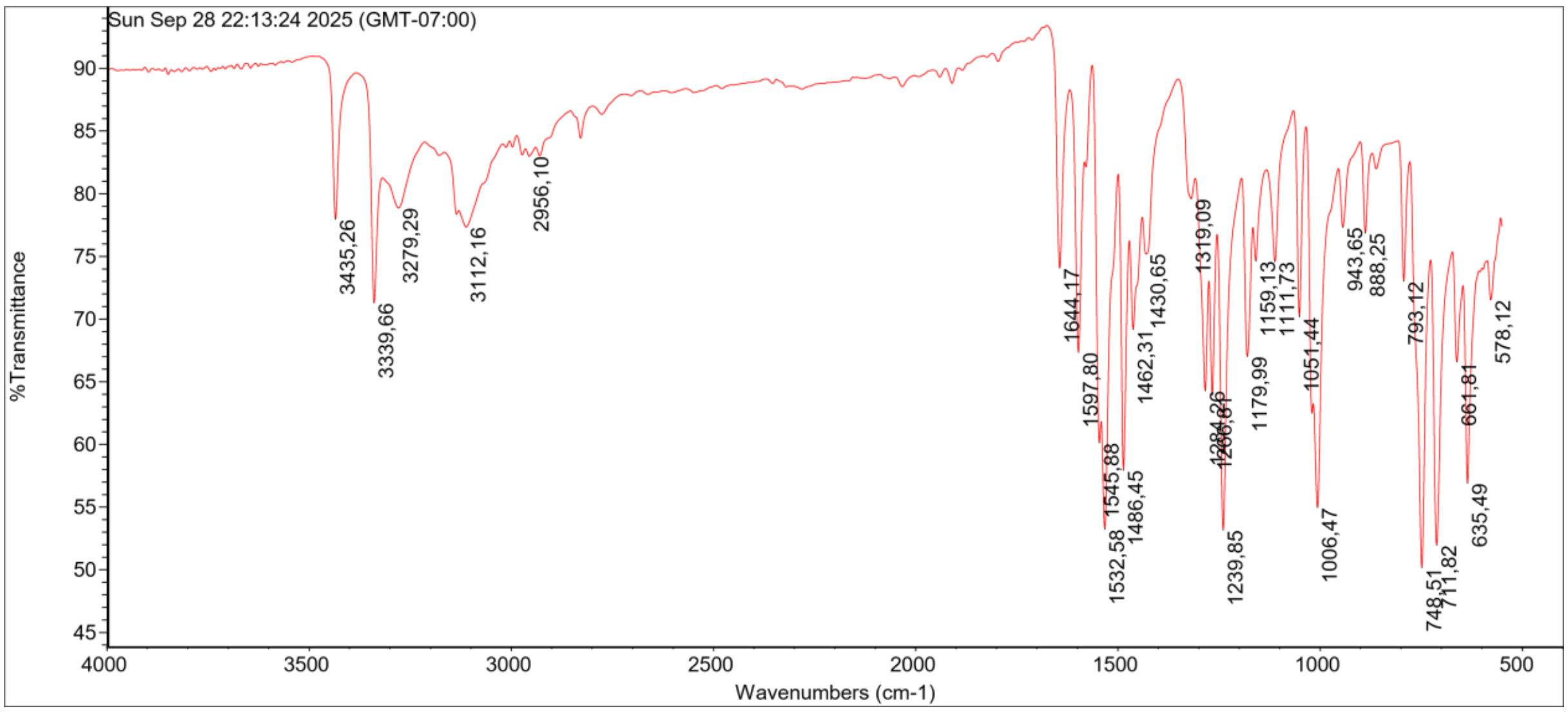

Figure S5. ATR-IR spectrum of 4-(2-methoxyphenyl)-1,3-selenazol-2-amine (compound **2**)

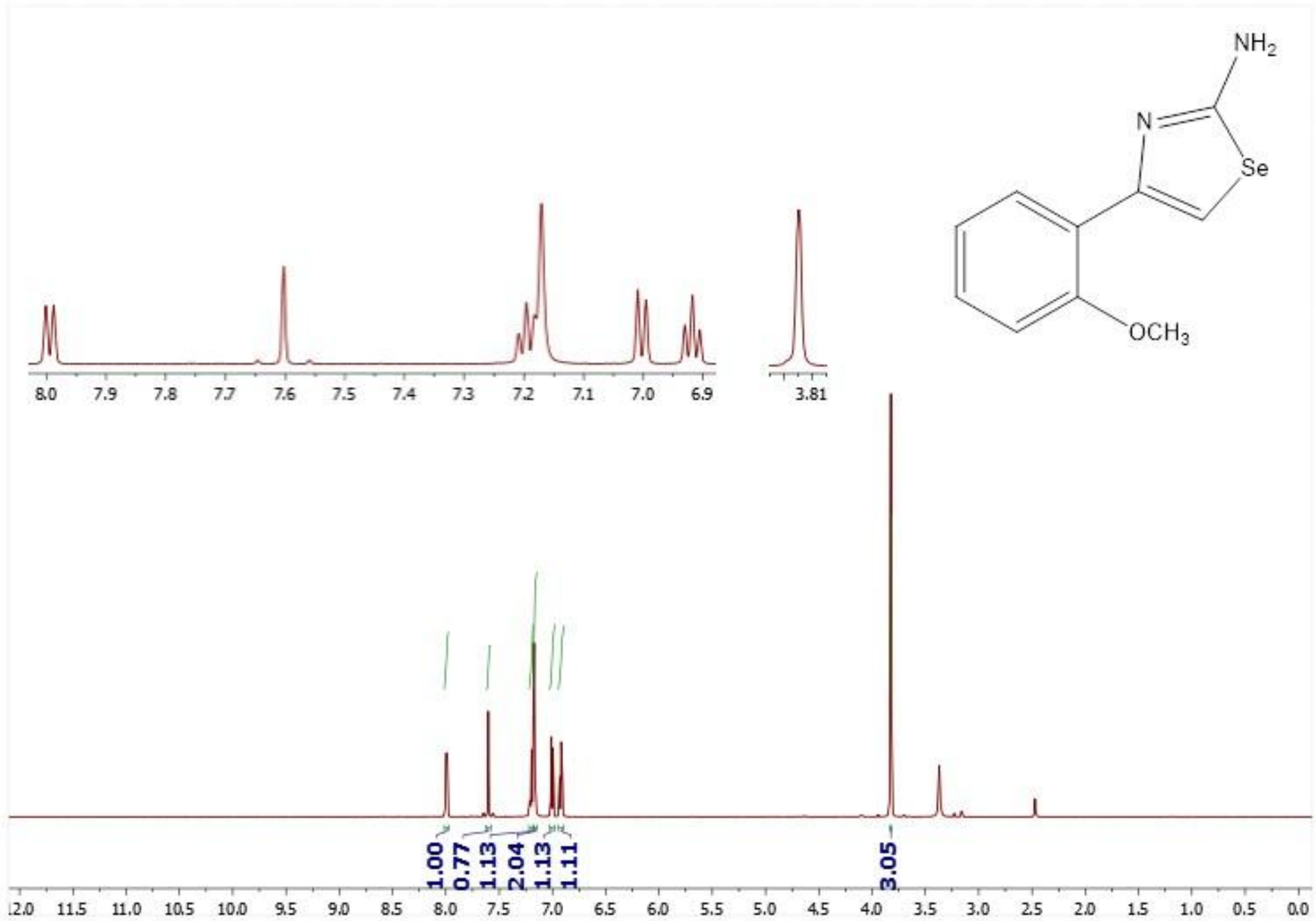

Figure S6. ^{1}H NMR spectrum of 4-(2-methoxyphenyl)-1,3-selenazol-2-amine (compound **2**)

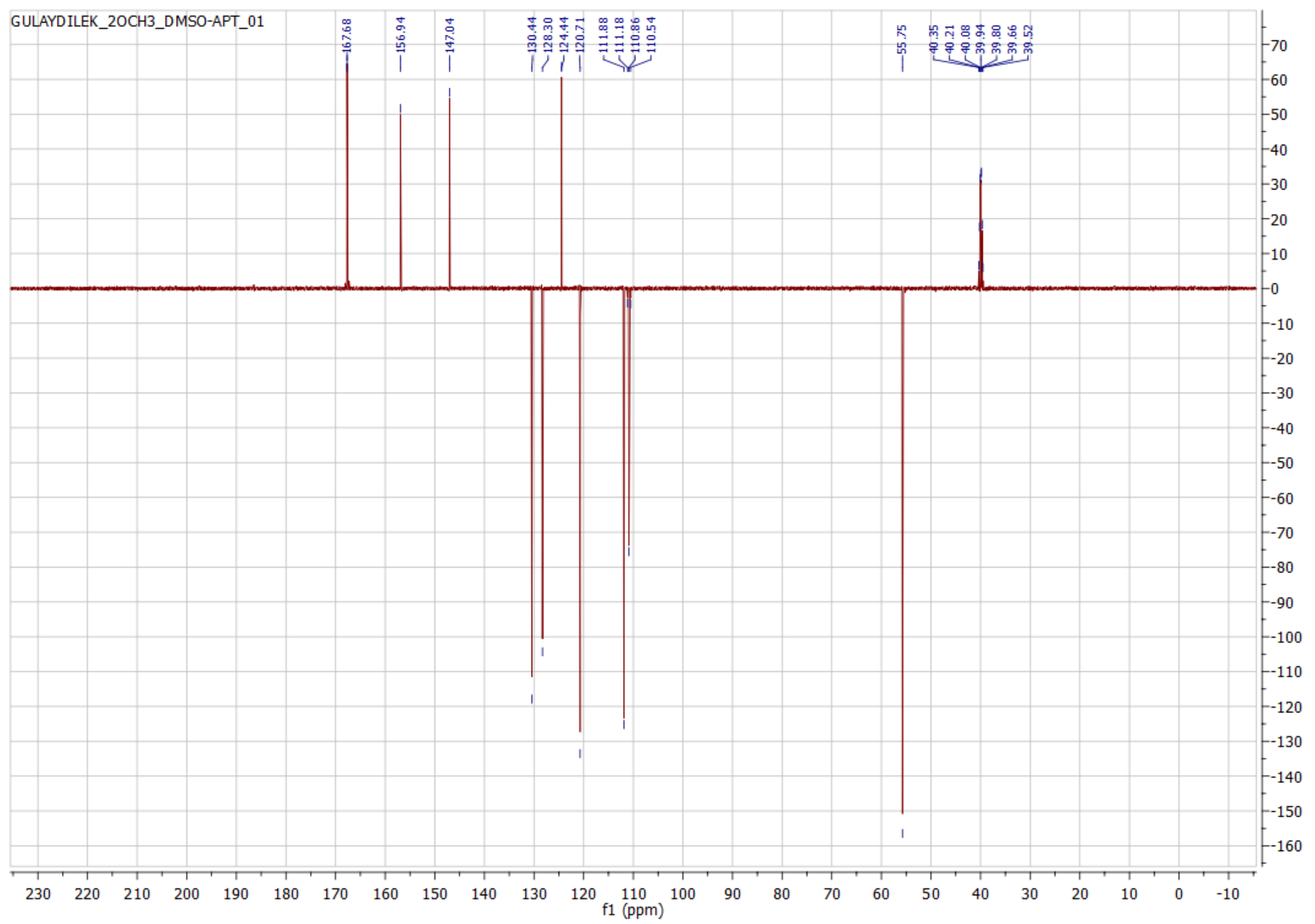

Figure S7. ^{13}C APT NMR spectrum of 4-(2-methoxyphenyl)-1,3-selenazol-2-amine (compound **2**)

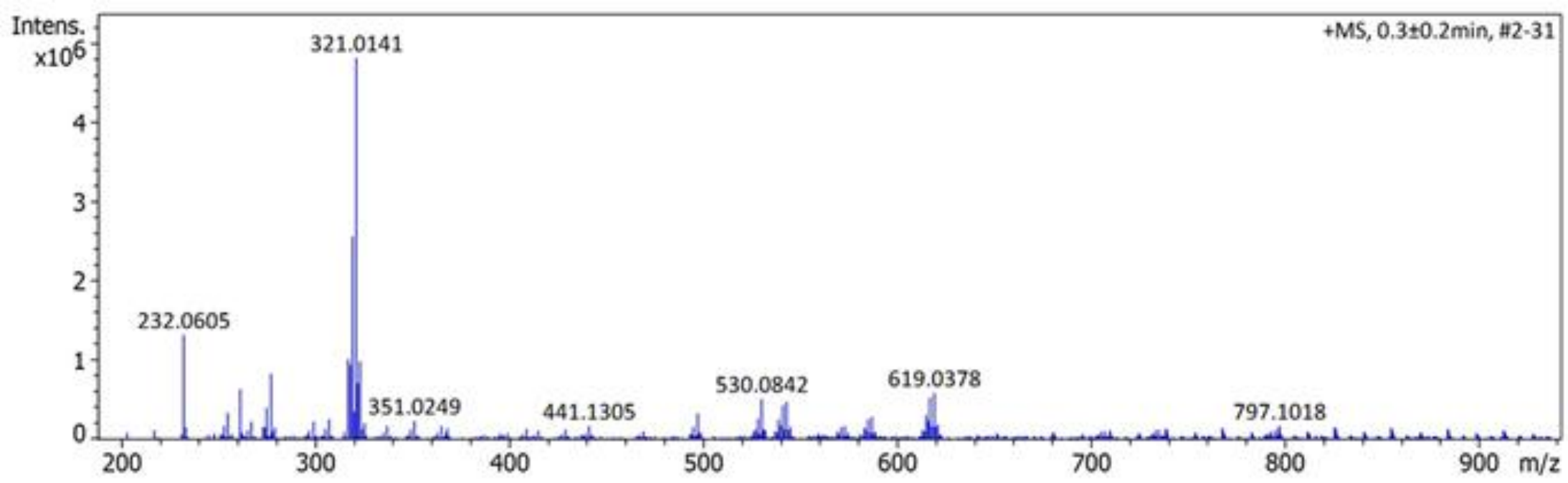

Figure S8. HRMS spectrum of 4-(2-methoxyphenyl)-1,3-selenazol-2-amine (compound **2**) recorded in positive electrospray ionization mode (ESI^+). The protonated molecular ion and sodium adduct were observed at *m/z* 255.0057 ($[M + H]^+$) and 276.9877 ($[M + Na]^+$), respectively.

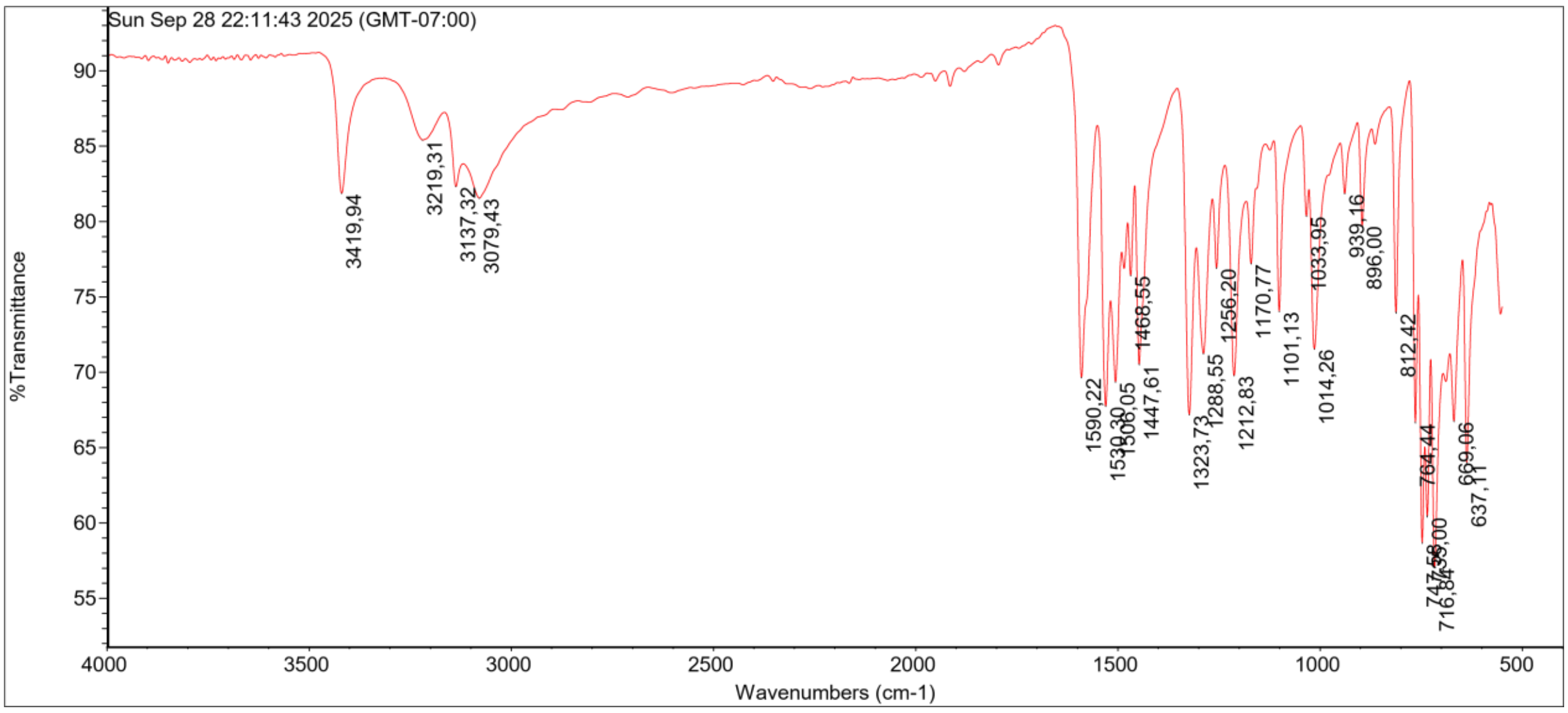

Figure S9. ATR-IR spectrum of 4-(2-fluorophenyl)-1,3-selenazol-2-amine (compound **3**)

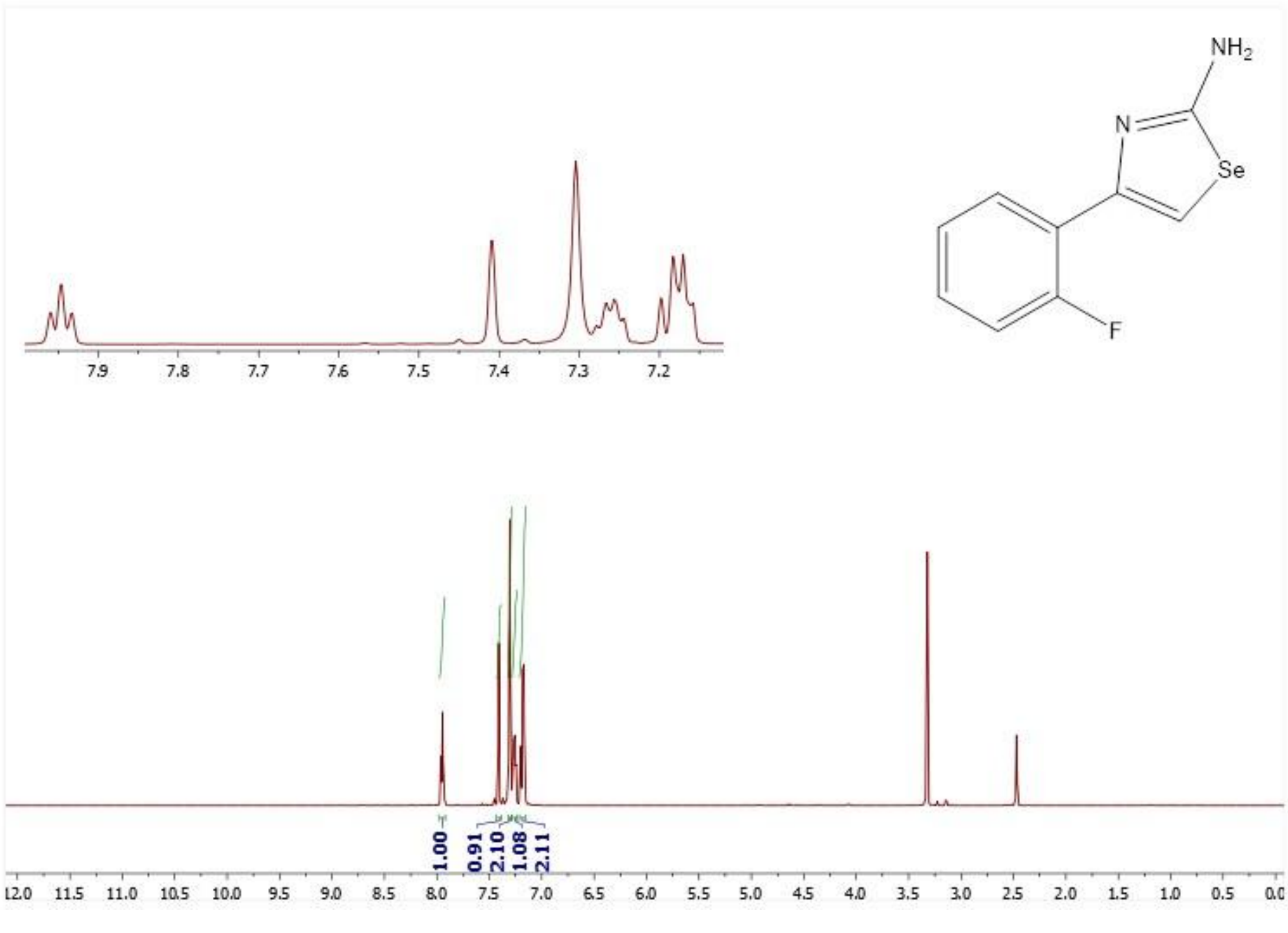

Figure S10. ^{1}H NMR spectrum of 4-(2-fluorophenyl)-1,3-selenazol-2-amine (compound **3**)

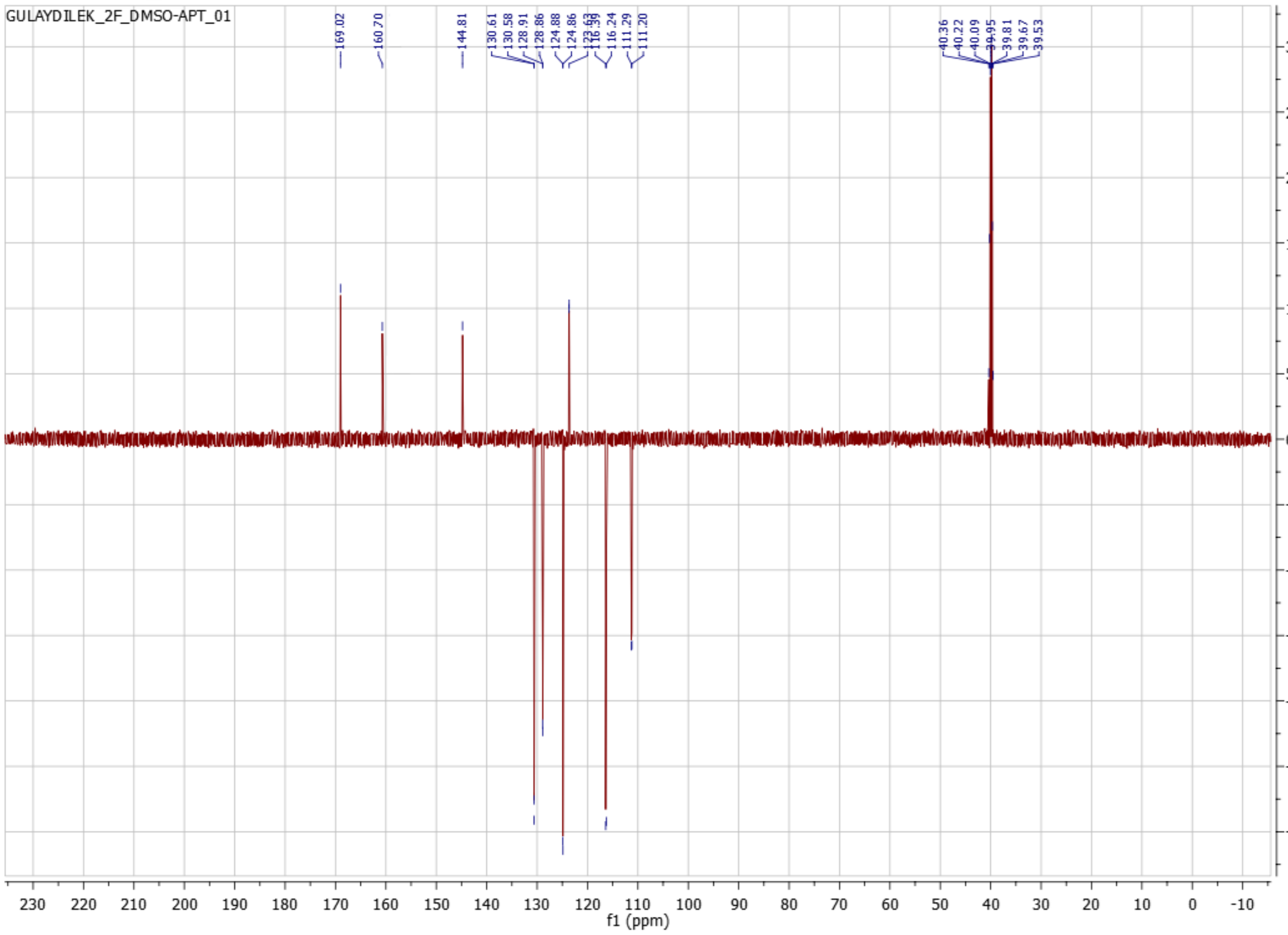

Figure S11. ^{13}C APT NMR spectrum of 4-(2-fluorophenyl)-1,3-selenazol-2-amine (compound **3**)

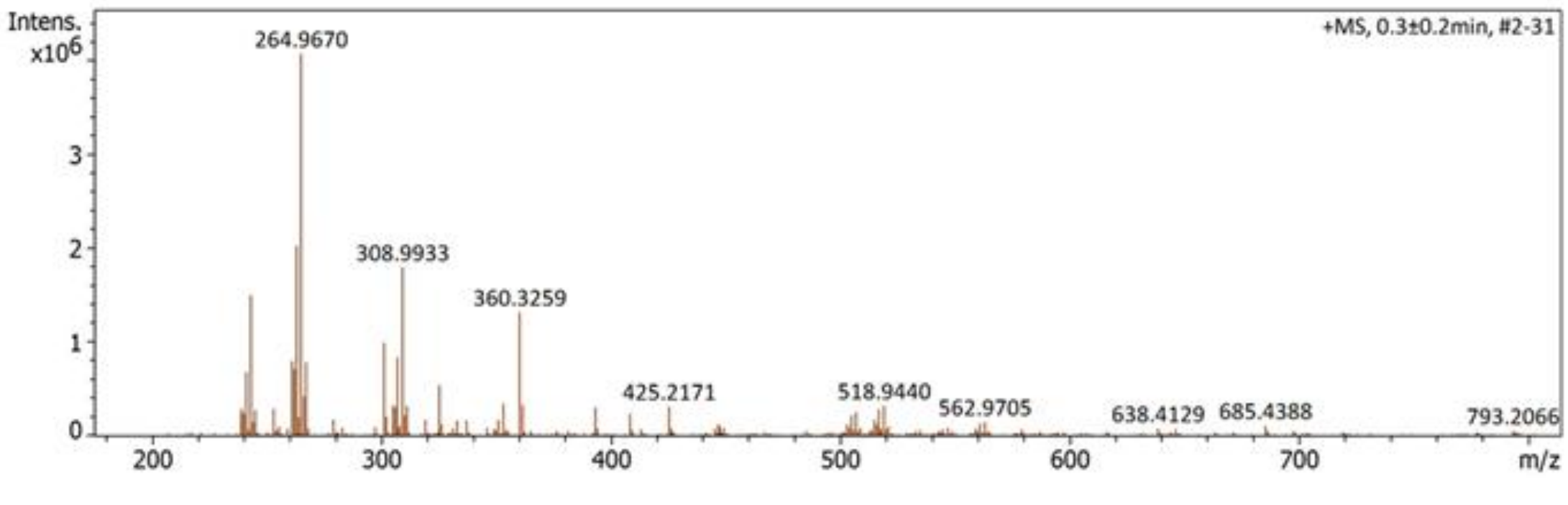

Figure S12. HRMS spectrum of 4-(2-fluorophenyl)-1,3-selenazol-2-amine (compound **3**)

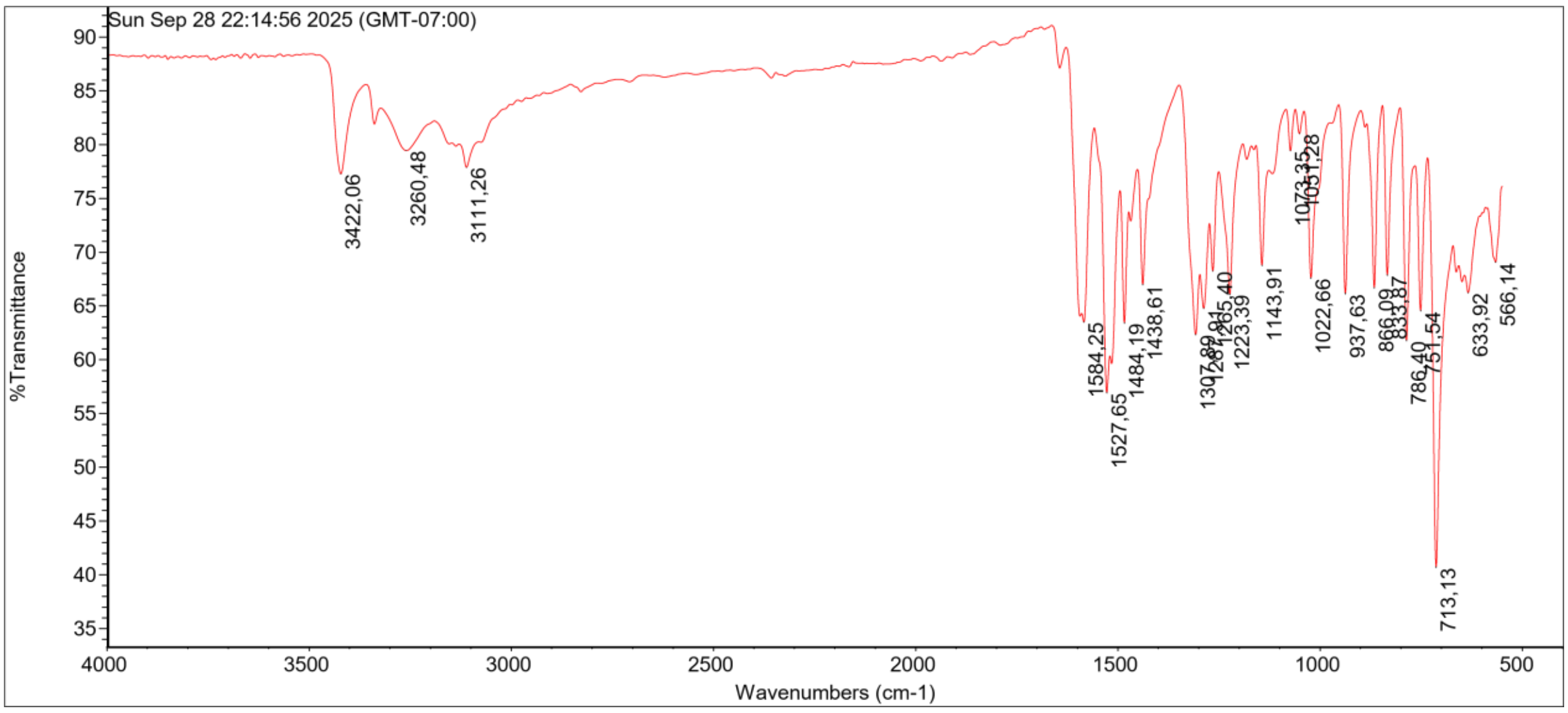

Figure S13. ATR-IR spectrum of 4-(3-fluorophenyl)-1,3-selenazol-2-amine (compound **4**)

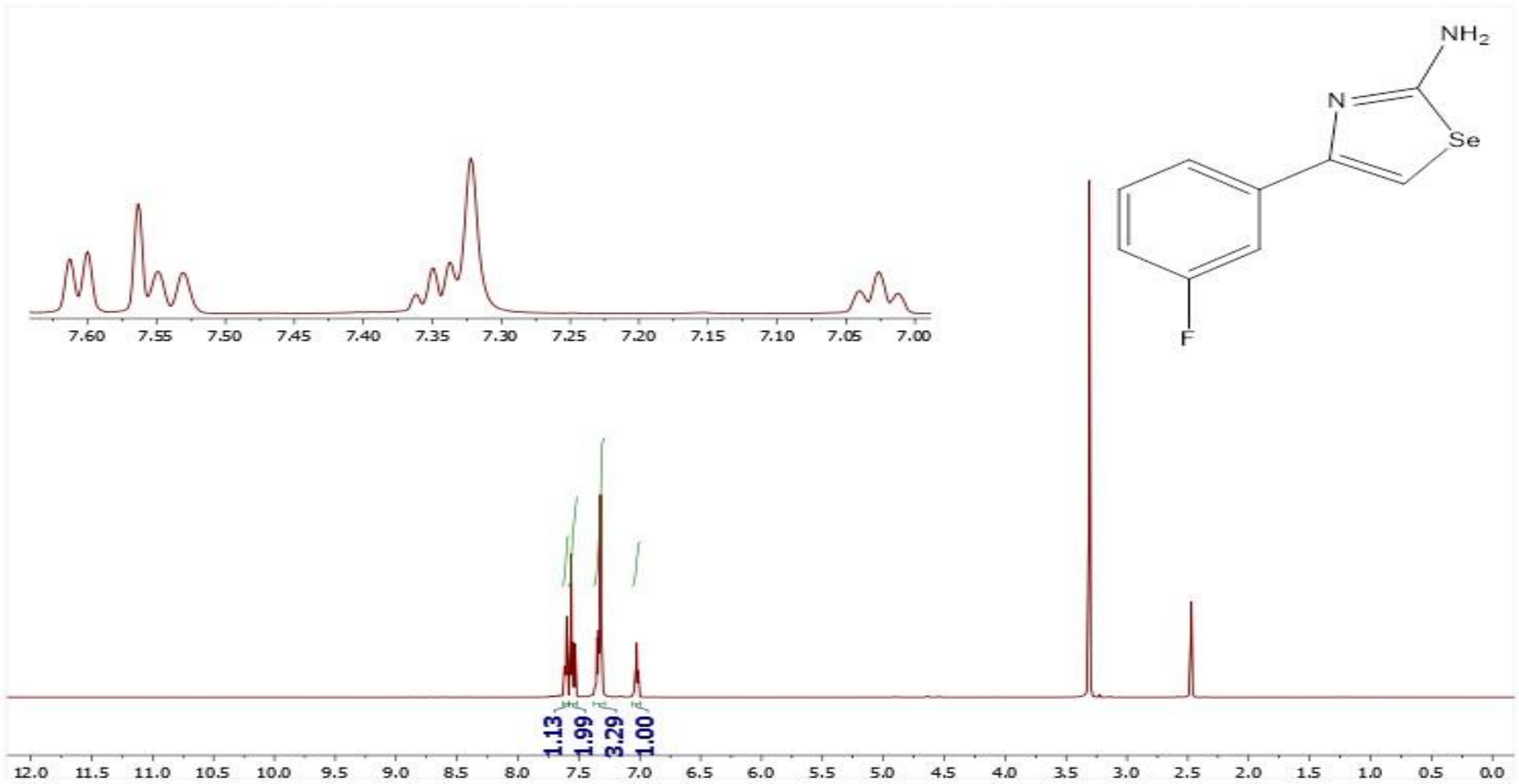

Figure S14. ^{1}H NMR spectrum of 4-(3-fluorophenyl)-1,3-selenazol-2-amine (compound **4**)

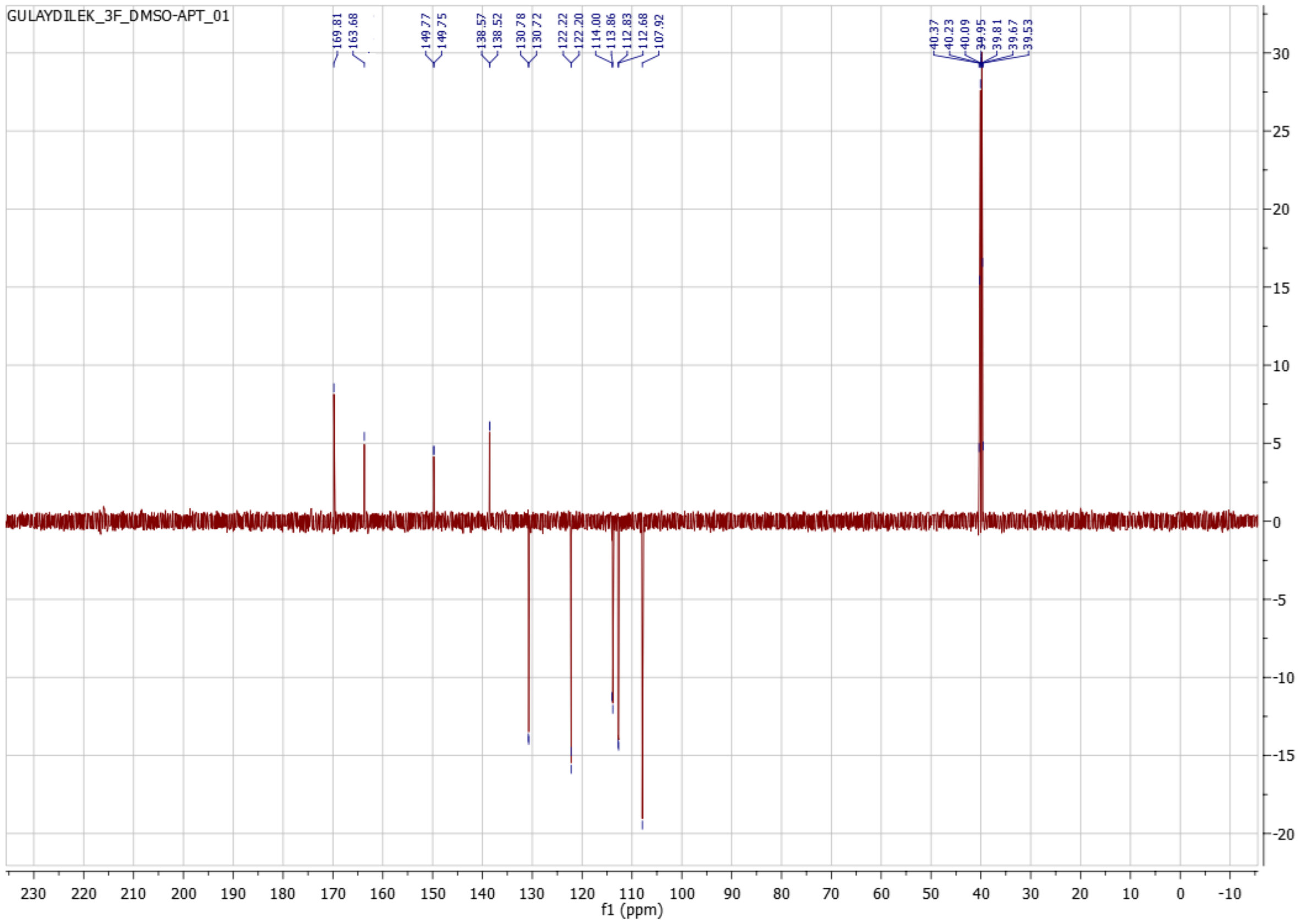

Figure S15. ^{13}C APT NMR spectrum of 4-(3-fluorophenyl)-1,3-selenazol-2-amine (compound **4**)

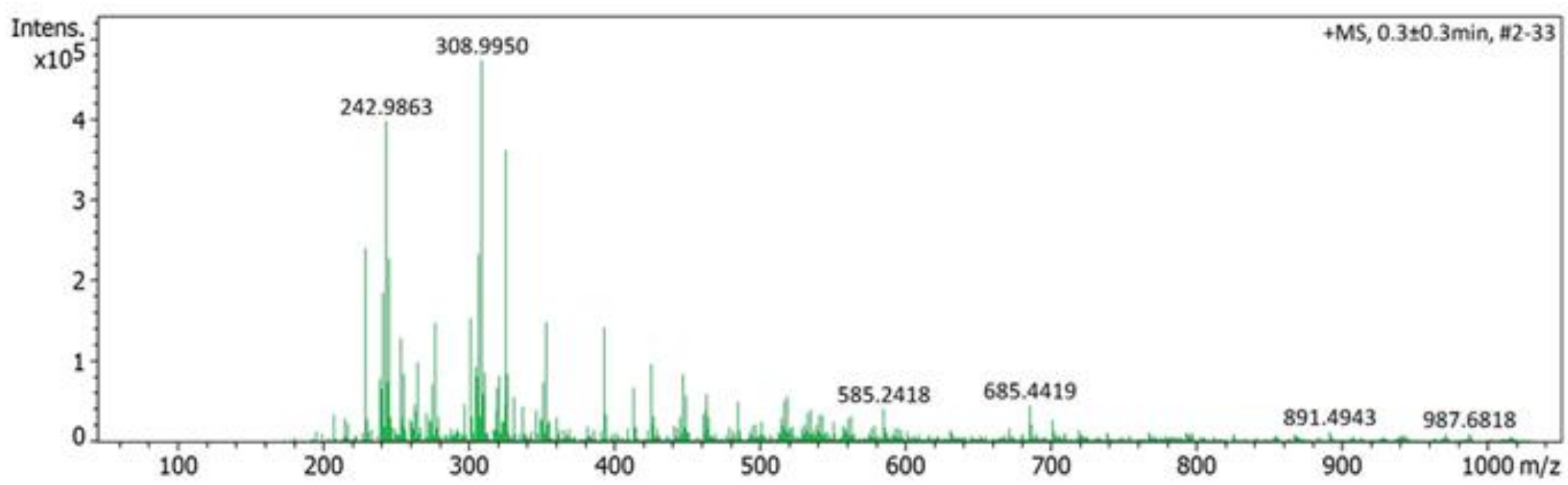

Figure S16. HRMS spectrum of 4-(3-fluorophenyl)-1,3-selenazol-2-amine (compound **4**)
